# Rare Variants Detection with Kernel Machine Learning Based on Likelihood Ratio Test

**DOI:** 10.1371/journal.pone.0093355

**Published:** 2014-03-27

**Authors:** Ping Zeng, Yang Zhao, Liwei Zhang, Shuiping Huang, Feng Chen

**Affiliations:** 1 Department of Epidemiology and Biostatistics, School of Public Health, Nanjing Medical University, Nanjing, Jiangsu, China; 2 Department of Epidemiology and Biostatistics, School of Public Health, Xuzhou Medical College, Xuzhou, Jiangsu, China; The University of Chicago, United States of America

## Abstract

This paper mainly utilizes likelihood-based tests to detect rare variants associated with a continuous phenotype under the framework of kernel machine learning. Both the likelihood ratio test (LRT) and the restricted likelihood ratio test (ReLRT) are investigated. The relationship between the kernel machine learning and the mixed effects model is discussed. By using the eigenvalue representation of LRT and ReLRT, their exact finite sample distributions are obtained in a simulation manner. Numerical studies are performed to evaluate the performance of the proposed approaches under the contexts of standard mixed effects model and kernel machine learning. The results have shown that the LRT and ReLRT can control the type I error correctly at the given *α* level. The LRT and ReLRT consistently outperform the SKAT, regardless of the sample size and the proportion of the negative causal rare variants, and suffer from fewer power reductions compared to the SKAT when both positive and negative effects of rare variants are present. The LRT and ReLRT performed under the context of kernel machine learning have slightly higher powers than those performed under the context of standard mixed effects model. We use the Genetic Analysis Workshop 17 exome sequencing SNP data as an illustrative example. Some interesting results are observed from the analysis. Finally, we give the discussion.

## Introduction

For next-generation sequencing data identifying rare variants associated with phenotypes of interest is both practically and theoretically important [1]–[3]. Here the rare variant is typically defined as allele with minor allele frequency (MAF) less than 1%. The past few years have witnessed increasing evidence that the rare variants play an important role in many complex diseases and disorders [4]–[16]. There are also some other findings supporting the contributions of rare variants to the diseases. For example, according to the odds ratio (OR) distribution, it has been demonstrated that most rare variants have values above 2 and the mean OR is 3.74, while very few common variants (defined as MAF>1%) have values above 2 and the mean OR is 1.36 [17]. See also Box 1 in Cirulli and Goldstein [2].

However, it is a very challenging task to detect the casual rare variants due to their extremely low MAF. For rare variant association analyses the single locus methods designed for common variants are rather underpowered or not applicable [1], [18]–[20], thus developing appropriate statistical approaches especially for rare variant has become an active research topic recently. A type of methods has been proposed by collapsing the rare variants within a functional region (e.g., gene and pathway) into one variant and then testing this collapsed variant [21]–[23]. In this paper, those tests are referred to as the burden test since they share the similar reasoning of collapsing. The burden test may be limited because it explicitly assumes that the variants within the collapsed region have the same direction of effect. However, in practice both protective and deleterious effects exist [1], [18], [19], [24]–[26].

More recently, Wu et al. [18] proposed the sequence kernel association test (SKAT) for rare variant detection. The SKAT is a score based variance component test originally developed by Lin [27] under the framework of mixed effects model [28], and has been widely applied to pathway or gene set analyses [29]–[32]. Two very attractive features of the SKAT are that: (I) it avoids the directionality of effect and consequently can enhance the statistical power when both protective and deleterious effects are present; (II) it proceeds under the framework of kernel machine learning, and thus can capture more complicated nonlinear relationship among rare variants.

The SKAT, however, has itself shortcomings as argued by Zhan and Xu [16]. For SKAT, a large score value (i.e., a small p value) does not necessarily mean the effect of a group of rare variants is also great, it may be due to a lot of variants with very weak effects. Additionally, when examining a set of rare variants, geneticists and epidemiologists may need some metrics to measure their contribution together, like OR in logistic regression or estimated coefficient in linear regression in single locus association analysis. While the SKAT will not involve any parameter estimation, thus cannot show effect differences across various sets of rare variants. Consequently, methods for rare variants with the capability to offer such information are desirable.

Motivated by the arguments above, in this paper we adopt the likelihood ratio test to detect the rare variants. Both the likelihood ratio test (LRT) and the restricted likelihood ratio test (ReLRT) are investigated and are performed under the same framework of mixed effects model of SKAT. A great advantage of LRT and ReLRT is that they not only examine the effect of a group of rare variants but also offer an effect measurement; this value in turn can be used to evaluate the relative importance of rare variants. To our best knowledge, the likelihood-based methods for rare variants have been not published before, nor are investigated under the framework of kernel machine learning, although the LRT and ReLRT are particularly popular in the literature.

In the rest of the paper, the SKAT and the burden test are first introduced, and then the LRT and ReLRT are discussed under the mixed effects model context and the kernel machine learning context, respectively. In this section, we will interpret how the kernel machine learning can be addressed with the mixed effects model and examine a group of rare variants via LRT and ReLRT. By using the eigenvalue representation of LRT and ReLRT, their exact finite sample distributions are obtained in a simulation manner. We perform extensive numerical studies to evaluate the performance of the proposed approaches and compare with the burden test and SKAT. The exome sequencing data from Genetic Analysis Workshop 17 (GAW17) is used as a practical application.

## Methods

### Notation

Let *X* = [*x*_1_, …, *x_p_*] denote the covariate vector of order *p* such as age, sex, smoking, and environmental exposure, and *G* = [*g*_1_, *g*_2_, …, *g_m_*] the genotype vector of order *m* for rare variants within a functional region specified a priori. In the paper, we use the additive genetic model, so that *g* = 0, 1, and 2 represent the number of minor alleles. For example, in the GAW17 data [33], [34], there are 16 single nucleotide polymorphisms (SNPs) included within the gene *KDR*, then the genotype can be expressed as *G* = [*g*_1_, *g*_2_, …, *g*_16_]. Let *Y* denote the continuous phenotype of interest (e.g., weight, blood pressure, and triglyceride) and *y_i_*, *i* = 1, 2, …, *n* its realization values, here *n* is the sample size. Suppose further that the phenotype *Y* follows a normal distribution with variance *σ* ^2^ conditional on the covariates *X* and genotypes *G*.

### Mixed effects model

First consider the linear mixed effects model [28], [35]

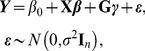
(1)where ***β*** = [*β*_1_, …, *β_p_*] are the fixed effects for covariates, *β*_0_ is the intercept, and **I** *_n_* is an identity matrix of order *n*; here ***γ*** = [*γ*_1_, …, *γ_m_*] are the random effects for rare genotypes, each *γ_j_*, *j* = 1, 2, …, *m* is assumed to be normally distributed with mean zero and variance *τw_j_* ^2^, where *τ* is a variance component and *w* is a prespecified weight related to MAF. For rare variant, *w* = Beta(MAF; 1, 25) is recommended in Wu et al [18], which places more weight on rarer variant and less weight on common variant, where Beta is the beta density function. In the present paper we also follow this idea, but make a slight modification. That is, a scaled weight of *w_j_ = w_j_*/max(***w***) is used, where the notation max indicates the maximum over all the *w_j_*s. In our experience, this modification is necessary to avoid numerical imprecisions encountered in the statistical software, such as the R statistical environment [36]. Greven et al. [37] gave a full description regarding this issue when performing the restricted likelihood ratio test for zero variance component in the linear mixed effects model.

Under these conditions, we can obtain
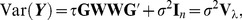
(2)where λ = *τ*/*σ* ^2^, 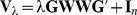, **W** is a diagonal matrix of order *m* with elements being *w*. Clearly testing whether or not a group of rare variants are collectively associated with the phenotype is equivalent to testing the null hypothesis *H*_0_: λ = 0. Note that the classical definition of heritability is defined as *τ*/(*τ+σ* ^2^), i.e., the proportion of phenotypic variance explained by a group of rare variants [38], then the heritability can be further expressed as λ/(1+λ). Therefore the quantity λ is an analogue of the heritability and can be employed for measuring the relative impotence of different groups of rare variants.

### Sequence kernel association test (SKAT)

According to Lee et al. [39] and Lee et al. [40], the original SKAT in Wu et al. [18] and the burden test can be studied within a unified framework if taking into account the correlation structure of the random effects. Suppose that the correlation structure among the *m* rare variants is **R** *_ρ_*, which is determined by the pairwise correlation coefficient corr(*g_j_*, *g_l_*) = *ρ* between any variants *j* and *l*. The unified SKAT statistic is given as
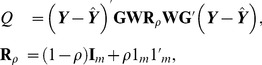
(3)where 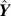 is the predicted value under H_0_. The test in Equation (3) is called the optimal SKAT (SKAT-O) since it can choose the correlation coefficient *ρ* adaptively to maximize the power when all the effects are in the same direction [39], [40].

When *ρ* = 0 (i.e., independent correlation), the SKAT-O reduces to the original SKAT in Wu et al. [18] and Lin [27], and when *ρ* = 1 (i.e., perfect correlation), the optimal SKAT reduces to the burden test.

Under *H*_0_, *Q* follows a mixture of chi-square distributions, the p values for the burden test and SKAT are obtained by the Davies method [41] or other methods [42], [43]. The p value for the SKAT-O is obtained by using a grid search strategy [39], [40].

### Likelihood ratio test (LRT) and restricted likelihood ratio test (ReLRT)

When examining variance component in the mixed effects model, the LRT and ReLRT are a natural alternative. Note that the null hypothesis *H*_0_: λ = 0 is non-standard since under *H*_0_ λ is on the boundary of the parameter space [44]–[47], and λ = 0 if and only if *τ* = 0. The parameter space for λ is Ω = [0, ∞).

Replacing ***γ*** and *σ* ^2^ in model (1) with their maximum likelihood (ML) estimators [47], we obtain the profile log-likelihood function up to a constant independent of the parameters
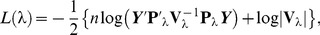
(4)where
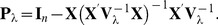
(5)The LRT statistic is defined as
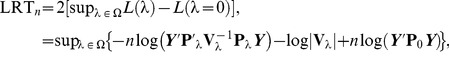
(6)where
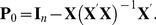
(7)Using the spectral representation [37], [47]–[49], it can be shown that LRT*_n_* is equal to the following quantity in distribution
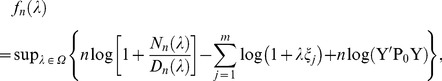
(8)where *ξ_j_*'s are the eigenvalues of matrix **W** ^1/2^ **G′GW** ^1/2^, and
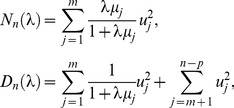
(9)where *μ_j_*'s are the eigenvalues of matrix **W** ^1/2^ **G′P**_0_ **GW** ^1/2^, and *u_j_*'s are independently standard normal random variables.

The ML estimator of *σ* ^2^ is biased downward since it does not take into account the loss in degrees of freedom due to estimation of ***γ***. While the restricted maximum likelihood (REML) method provides an unbiased estimator for *σ* ^2^ by using a set of *n* - *p* linearly independent error contrasts [50]–[53]. The profile restricted log-likelihood function up to a constant independent of the parameters is given as
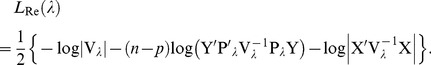
(10)The ReLRT statistic is defined as
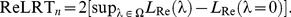
(11)


Using the similar reasoning for LRT*_n_*, it can be shown that ReLRT*_n_* is equal to
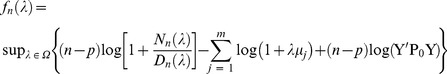
(12)in distribution.

By taking full advantage of the spectral representation used in Equations (8) and (12), Crainiceanu and Rupper [47] described a simulation-based algorithm for the finite sample distributions of LRT*_n_* and ReLRT*_n_*. This algorithm has been shown to be rather fast and accurate. The p values of the LRT and ReLRT are obtained by comparing the observed statistics to those simulated values.

### Kernel machine learning

So far we have discussed how to detect the causal rare variants by using the LRT and ReLRT which are developed under the standard mixed effects model context. In this section, we turn to the recently popular kernel machine learning, explore its relationship with the mixed effects model, and demonstrate how to detect the causal rare variants in the kernel machine learning context via LRT and ReLRT. As we will see, there is a close connection between these two statistical theories, which provides a more flexible way for rare variant detection with kernel methods.

Using the same notation defined before, we describe the relationship between the phenotype *Y* and genotypes *G* and covariates *X* via a semi-parametric linear model [30], [31]

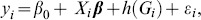
(13)where *h* is an unknown smooth function lying in a Hilbert space *Η_K_* generated by a positive definite kernel function *K* [31], [54]. This space is called reproducing kernel Hilbert space (RKHS) under some regularity conditions [55]–[58]. The kernel function *K* essentially quantifies the genomic similarity or distance of two subjects and can be arbitrarily chosen as long as it satisfies the conditions of Mercer's theorem [55], [57]. Model (13) is semi-parametric since the covariates *X* are fitted parametrically while the genotypes *G* are fitted non-parametrically.

To avoid over-fitting, estimation of *h* can be performed by maximizing the penalized log-likelihood function [31], [59]

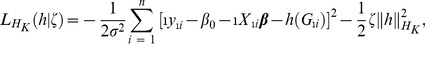
(14)where *ζ* is a penalization parameter controlling the balance between the goodness of fit and the complexity of the model [31], [59], and the notation ∥·∥ is the norm in RKHS. The solution of *h* in Equation (14) is given in terms of the well-known representer theorem of Kimeldorf and Wahba [60] and Wahba [61]

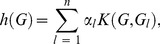
(15)where ***α*** = [*α*_1_, *α*_2_, …, *α_n_*] is an unknown vector of parameters and *K* is a reproducing kernel function [31], [54].

We further rewrite *h* in the form of matrix as
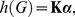
(16)where **K** is an *n*×*n* kernel matrix with its elements being *K*(*G_i_*, *G_l_*). Various kernel functions have been designed in genetic statistics [59], [62], such as the linear kernel, the polynomial kernel, the Gaussian kernel, and the identify by state (IBS) kernel. The explicit forms for these kernels can be found in Wu et al. [18], Wu et al. [32], Liu et al. [31], Kwee et al. [29], and Liu et al. [30]. If a kernel is weighted, then it is called a weighted kernel. In the paper the scaled weight described in Section 2.2 is used. Additionally, once the kernel function is chosen, we assume that **K** is known completely. Consequently, inference about *h* in model (14) immediately reduces to inference about ***α***.

Replacing *h* in Equation (14) with (16) yields
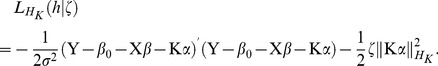
(17)Following the results of Gianola et al. [54], Wahba [61], and Wahba [63],
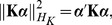
(18)
Equation (17) is re-expressed as
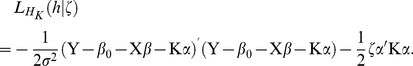
(19)From a Bayesian perspective [31], [59], Equation (19) is the log-posterior distribution of *β*_0_, ***β*** and *α*, thus can be described as the following hierarchical model
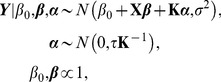
(20)where *τ* = 1/*ζ*. Since *h* = **Kα**, alternatively the hierarchical model is re-expressed as
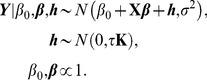
(21)In the paper we use the hierarchical model (21) since it avoids the calculation of inverse matrix and therefore reduces the computational cost.

Based on the arguments described above, we can construct the relationship between the semi-parametric linear model (13) and the mixed effects model (1). That is, model (13) is equivalent to the following mixed effects model
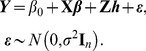
(22)The differences between model (22) and model (1) mainly lie in two aspects: (I) here **Z** is an identify matrix of order *n*, while **G** in model (1) is of dimension *n*×*m*; (II) the unknown parameter ***h*** here is an *n*-dimensional vector with its covariance-variance matrix being *τ* **K**, while in model (1) the unknown parameter ***γ*** is an *m*-dimensional vector with its covariance-variance matrix being diag(*τw_j_* ^2^), here the notation diag indicates a diagonal matrix.

Therefore, all the theories for the LRT and ReLRT developed under the context of mixed effects model can be also applicable in the context of kernel machine learning. The test of variance component in model (22) can proceed similarly in model (1). To distinct these two types of approaches, in the reminder of the paper, LRT.M and ReLRT.M are used to indicate the LRT and ReLRT for the mixed effects model, LRT.K and ReLRT.K are used to indicate the LRT and ReLRT for the kernel machine learning, and LRT and ReLRT are used to indicate both the two types.

## Results

### Simulation datasets

We generate genotypes based on the coalescent model for European population by using the package COSI [64]. A total of 100 kb gene region is simulated. Randomly selected continuous 30% subregions of the simulated genotypes are used. Variants with MAF less than 0.01 are defined as rare variants. Two covariates are considered, *x*_1_ is a standard normal variable and *x*_2_ is a binary variable with rate 0.5, and mutually independent. The sample size *n* is 300, 400, and 500.

For type I error simulations the phenotype is generated as
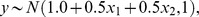
and the number of runs is 2,000. In power simulations, 30% rare variants are causal variants, the effect size |*γ*| is 0.3|log_10_MAF|, which leads to a size of 1.2 for MAF = 0.0001 and a size of 0.6 for MAF = 0.01. Among the causal rare variants, 0%, 30% or 50% have negative effects, i.e., in these settings their effects are −0.3|log_10_MAF|. For power simulations the phenotype is generated as
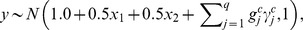
where *q* is the number of chosen causal rare variants, *g^c^*'s are the genotypes and *γ^c^*'s are the effect sizes given above. The number of runs is 1,000. The simulation characteristics under these specifications are displayed in **Table** 1.

**Table 1:** Simulation characteristics.

*n*	Total SNPs	Selected SNPs	Used rare variants	Causal rare variants
300	417	125	41	12
400	434	130	47	14
500	447	134	51	15

In the present paper, seven methods including burden test, SKAT, SKAT-O, LRT.K, ReLRT.K, LRT.M, and ReLRT.M are compared. The first three tests are performed in the package SKAT [18], and the LRT and ReLRT are performed in the package RLRsim[65]. In practice the weighted kernel has been empirically shown to be more powerful compared to its unweighted counterpart [18], [29], thus here we only consider the former. For comparison only the weighted linear kernel is used since under this situation both the mixed effects models in (1) and (22) are well specified, and the burden test and the SKAT-O are only able to be performed on the linear kernel.

### Type I error and power

**Figure 1:**
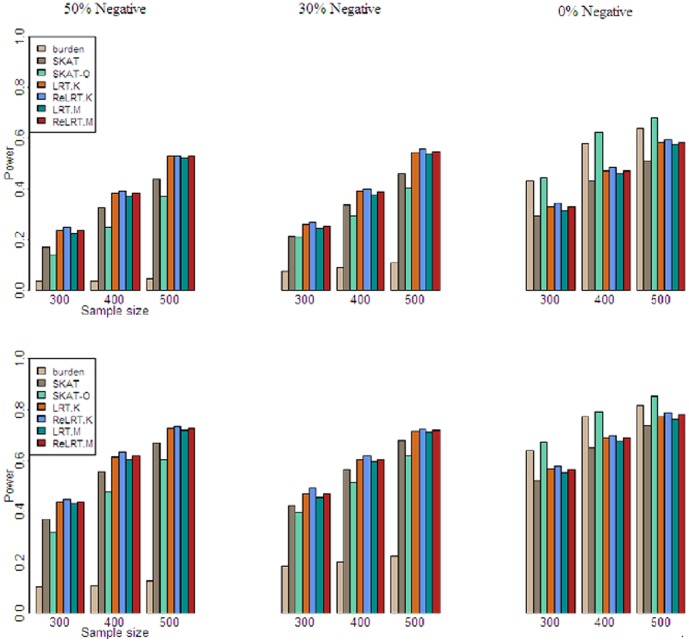
Estimated power for all the tests. The top panel is for *α* = 0.01 and the bottom panel is for *α* = 0.05. M% negative means that in these associated SNPs M% have effects −0.3|log_10_MAF| and the rest (100-M)% are 0.3|log_10_MAF|.

**Table** 2 displays the estimated Type I errors for all the tests. It can be seen from **Table** 2 that all the tests control the type I error correctly at the given *α* level. **Figure** 1 shows the estimated powers. **Table**s 3 and 4 present the losses of power for the situation that 30% or 50% causal rare variants have negative effects compared to the situation that none of the causal rare variants has negative effects. These values are obtained according to **Figure** 1. The average values in **Table**s 3 and 4 are calculated across sample sizes.

**Table 2:** Estimated Type I error.

*n*	Burden	SKAT	SKAT-O	LRT.K	ReLRT.K	LRT.M	ReLRT.M
		*α* = 0.05					
300	0.051	0.042	0.043	0.047	0.052	0.041	0.046
400	0.060	0.044	0.055	0.050	0.056	0.048	0.051
500	0.058	0.043	0.054	0.042	0.046	0.040	0.042
		*α* = 0.01					
300	0.010	0.008	0.008	0.011	0.012	0.010	0.010
400	0.012	0.008	0.012	0.010	0.012	0.010	0.010
500	0.010	0.008	0.010	0.010	0.010	0.009	0.010

**Table 3:** Losses of the power for *α* = 0.05&.

*n*	burden	SKAT	SKAT-O	LRT.K	ReLRT.K	LRT.M	ReLRT.M
		30% Negative#					
300	0.456	0.100	0.280	0.098	0.087	0.099	0.096
400	0.574	0.089	0.279	0.086	0.079	0.081	0.084
500	0.593	0.059	0.233	0.058	0.065	0.052	0.063
Average$	0.541	0.083	0.264	0.081	0.077	0.077	0.081
		50% Negative#					
300	0.537	0.151	0.356	0.128	0.132	0.124	0.127
400	0.667	0.096	0.313	0.073	0.066	0.073	0.071
500	0.692	0.068	0.247	0.050	0.052	0.043	0.053
Average$	0.632	0.105	0.305	0.084	0.083	0.080	0.084

& : The values are differences of power between the situation with none of the causal variants (i.e., 0%) being negative and the situation with 30% or 50% causal variants being negative.

# : It means that 30% or 50% causal variants are negatively related to phenotype with effects −0.3|log_10_MAF| and the rest 70% or 50% are positively related to phenotype with effects 0.3|log_10_MAF|.

$ : The average is calculated across sample sizes.

**Table 4:** Losses of the power for *α* = 0.01&.

*n*	burden	SKAT	SKAT-O	LRT.K	ReLRT.K	LRT.M	ReLRT.M
		30% Negative#					
300	0.355	0.080	0.233	0.072	0.078	0.068	0.074
400	0.490	0.092	0.330	0.081	0.086	0.083	0.083
500	0.530	0.052	0.279	0.039	0.038	0.038	0.036
Average$	0.458	0.075	0.281	0.064	0.067	0.063	0.064
		50% Negative#					
300	0.393	0.124	0.301	0.094	0.094	0.090	0.091
400	0.545	0.105	0.373	0.089	0.092	0.090	0.087
500	0.592	0.074	0.310	0.054	0.065	0.054	0.056
Average$	0.510	0.101	0.328	0.079	0.084	0.078	0.078

& : The values are differences of power between the situation with none of the causal variants (i.e., 0%) being negative and the situation with 30% or 50% causal variants being negative.

# : It means that 30% or 50% causal variants are negatively related to phenotype with effects −0.3|log_10_MAF| and the rest 70% or 50% are positively related to phenotype with effects 0.3|log_10_MAF|.

$ : The average is calculated across sample sizes.

Some important observations from **Figure** 1, **Table**s 3 and 4 are listed as follows.

1. When all the causal rare variants have the same direction of effect, the burden test and the SKAT-O are the most powerful, following by the LRT, ReLRT, and SKAT. These results are expected since both the burden test and the SKAT-O are designed especially for this situation.
2. When both positive and negative effects are present, all the tests suffer from power decrease. Under this situation, the LRT and ReLRT have the highest powers, and the burden test suffers from the most reduction of power. For example, when *α* = 0.05, *n* = 500 and all causal rare variants are in the same direction, for the burden test its power is 0.817, while its power decreases to 0.224 when 30% causal rare variants have negative effects and 0.125 when 50% causal rare variants have negative effects. The SKAT-O is no longer optimal and has a smaller power compared to the SKAT, suggesting that in practice using the SKAT rather than the SKAT-O may be safer since the situation that positive and negative effects occur simultaneously is more frequent than the situation that all the effects are in the same direction. Compared with the SKAT, the LRT and ReLRT reduce fewer powers, implying these two tests are relatively more robust to the mixture effects of rare variants.
3. It can be seen that the LRT and ReLRT consistently outperform the SKAT regardless of the sample size and the proportion of the negative causal rare variants.
4. The ReLRT always has a higher power than the LRT, which may stem from the fact that the ReLRT gives the unbiased estimator of variance component.
5. The LRT.K versus LRT.M and the ReLRT.K versus ReLRT.M behave comparably, but it is interesting that the ReLRT.K has a slightly larger power than the ReLRT.M, and the LRT.K also has a slightly larger power than the LRT.M.

### Application

We apply these methods to the unrelated samples of the GAW17 [33], [34]. The GAW17 data contains 24,487 SNPs across 3,205 autosomal genes on 697 individuals, three covariates (age, sex and smoke), three quantitative traits (Q1, Q2 and Q4), and a binary trait. Most of the SNPs are rare with MAF ranging from 0.07% to 25.8%, 74% have MAF less than 0.01 and 12.8% have MAF more than 0.05. This data was widely used on Genetic Analysis Workshop 17 to evaluate the newly developed methods for rare variant detection and compare to the existing ones.

Here we choose the quantitative trait Q1, and select the SNPs within genes *HIF3A*, *FLT1* and *KDR*. These selected genes are rather typical for our comparison of the methods. For *HIF3A*, 20% SNPs are causal rare variants with weak effects. For *FLT1*, 44% SNPs are causal rare variants with moderate effects. For *KDR*, 71.4% SNPs are causal rare variants with relatively strong effects. The characteristics of the selected data are depicted in **Table** 5. More detailed information regarding GAW17 data can be found in Almasy et al. [34].

**Table 5:** Characteristics of the used GAW17 data#.

Gene	Chr	Total	Rare	Causal	MAF	Causal Effects
*HIF3A*	19	21	15	3	7.17×10^−3^∼0.385	0.174668, 0.51468, 0.265181
*FLT1*	13	35	25	11	7.17×10^−3^∼0.291	0.18047, 0.457361, 0.732566, 0.839669, 0.38582, 0.549816, 0.623466, 0.653351, 0.59670, 0.549214, 0.090586
*KDR*	4	16	14	10	7.17×10^−3^∼0.165	0.598271, 0.715613, 0.503025, 1.17194, 0.149975, 0.610938, 0.318125, 0.312058, 1.171940, 0.417977

# : Chr indicates the chromosome, Total indicates the total number of SNPs contained in the gene, and Rare indicates the number of rare SNPs within the gene.

We use the weighted linear kernel and define the rare variant as those with MAF less than 0.01, so the SNPs with MAF greater than such cut point are not included in the analysis. The results are listed in **Table** 6. The two types of LRT and ReLRT lead to the same results; to save space only one type is reported.

**Table 6:** Results of the used GAW17 data.

			p value				λ
Gene	Burden	SKAT	SKAT-O	LRT	ReLRT	LRT	ReLRT
*HIF3A*	0.262	0.483	0.420	0.388	0.387	<0.001	<0.001
*FLT1*	6.12×10^−8^	9.01×10^−7^	1.03×10^−9^	6.28×10^−7^	5.44×10^−7^	0.750	0.748
*KDR*	9.27×10^−7^	1.29×10^−3^	2.78×10^−6^	4.99×10^−5^	4.83×10^−5^	1.778	1.767

Some interesting results are observed form **Table** 6.

1. Since all the causal rare SNPs within each gene are positively related to the phenotype Q1 [34], the burden test and SKAT-O have the smallest p values compared to other methods. The LRT and ReLRT obtain smaller p values than SKAT, and the ReLRT always has smaller p values compared to LRT.
2. Due to the weak effects and small proportion of rare variants, the *HIF3A* cannot be discovered by all the methods; while the *FLT1* and *KDR* are successfully detected. But here it is noted that the p value of SKAT (1.29×10^−3^) for *KDR* is much larger than those of LRT and ReLRT (with scale of 10^−5^).
3. The burden test, SKAT, and SKAT-O cannot give any evidence regarding the effect of the gene. For instance, *FLT1* and *KDR* can be viewed as moderate and strong signals, respectively, but instead the former has a much smaller p value than the latter. This may show a mistaken impression that the *FLT1* is more associated with the phenotype. Fortunately, the estimates of λ provided by LRT and ReLRT display the distinction, that is, the value of λ for *KDR* is larger than that for *FLT1*. From **Table** 6, it can be seen that the estimates of λ correctly reveal the effect strength of different genes. Here the result empirically documents that the LRT and ReLRT are preferred to the SKAT when comparing the contributions of various genes based on a set of rare variants.

## Discussion

In this paper we have proposed the LRT and ReLRT to detect the rare variants associated with complex phenotypes from both the standard mixed effects model framework and the kernel machine learning context. In the latter, the original space of genotypes is mapped to another higher dimensional space by the kernel function. Such a space may be potentially infinite dimensional and is referred to as a feature space in the machine learning literature where the model can proceed linearly [55], [57], [66]. An important advantage of kernel methods is that we do not have to construct the feature space explicitly since all the analyses can be finished directly over the kernel [66]. In fact the kernel function itself is frequently more efficient to compute than the map function or the inner product induced in *Η_K_* [55], [57].

By using the representer theorem, the connection between the kernel machine learning and the mixed effects model is well established from the Bayesian point of view. This connection provides a convenient way to examine the rare variants under the context of kernel methods using the LRT and ReLRT. We can find that the kernel is actually the covariance structure for the random effects *h*, so it can be thought to be the prior correlation among subjects.

Our simulations have demonstrated that the performance of the LRT and ReLRT in the two contexts is comparable. However, it can be expected that the methods of LRT.K and ReLRT.K should be more flexible and attractive although only the linear kernel function is employed in the paper; but even then the LRT.K and ReLRT.K have displayed slightly larger powers than the LRT.M and ReLRT.M. Extending the proposed LRT and ReLRT to other kernel functions needs no any additional efforts, but more applications in practice are required to further understand the behaviors of various kernels. The choice of a kernel function is dependent on which feature space is used to approximate *h* [30], [31]. Liu et al. [31] showed that in a simulation example the Gaussian kernel performed the best compared to other competing kernels.

In the paper, the exact finite sample distributions of LRT and ReLRT obtained by simulation are employed. One may attempt to use the 50∶50 mixture distribution of 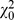 and 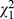 [44]–[46], where 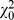 is a point probability mass at zero and 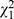 is a chi-square distribution with 1 degree of freedom. However, it has been displayed that this mixture distribution is conservative [37], [47]. It is obvious that the application of the exact finite sample distribution improves the powers of LRT and ReLRT. In addition, the LRT and ReLRT are required to estimate both the null and alternative models. By doing this more information especially from the rare variants is incorporated into the tests, accordingly the powers increase.

Our simulations have also demonstrated that the LRT and ReLRT (including LRT.K and ReLRT.K, and LRT.M and ReLRT.M) outperform the SKAT regardless the sample size and the proportion of negative effects of rare variants. Consequently our results here offer some empirical evidence that the LRT and ReLRT may be preferable to the score test (i.e., the SKAT) in the case of finite sample where the parameter of interest is constrained on the boundary. See also Kuo [48] and Verbeke and Molenberghs [67].

In this paper there are some other aspects concerning the kernel machine learning in rare variant detection that is warranted to be explored. For example, how to choose an optimal kernel function for real life sequencing data [31], [68], how to select substantially important random effects (i.e., the true subset of rare variants associated with the phenotype) in a kernel function [69], and what are the exact finite sample distributions of the LRT and ReLRT if incorporating tuning parameters into the kernel function as done in Mallick et al. [58] and Liu et al. [31]. These problems are certainly interesting topics for further investigations.
